# Synergistic alleviation effects of salt-tolerant plant growth-promoting rhizobacteria and hydrogen-rich water on salt stress in *Pennisetum giganteum*

**DOI:** 10.3389/fpls.2025.1702577

**Published:** 2025-10-29

**Authors:** Shaohua Chu, Ting Xu, Yuying Feng, Xianzhong Ma, Ruitian Shu, Renyuan Wang, Yu Wang, Doudou Jin, Yaowei Chi, Pei Zhou, Dan Zhang

**Affiliations:** ^1^ School of Agriculture and Biology, Shanghai Jiaotong University, Shanghai, China; ^2^ Key Laboratory of Urban Agriculture, Ministry of Agriculture and Rural Affairs, Shanghai, China; ^3^ Shanghai Yangtze River Delta Eco-Environmental Change and Management Observation and Research Station, Shanghai, China; ^4^ Shanghai Urban Forest Ecosystem Research Station, National Forestry and Grassland Administration, Shanghai, China; ^5^ Bor S. Luh Food Safety Research Center, Shanghai, China; ^6^ Chongming Agricultural Environment Field Science Observation and Research Station, Ministry of Agriculture and Rural Affairs, Shanghai, China; ^7^ Yunnan Dali Research Institute, Shanghai Jiaotong University, Shanghai, China

**Keywords:** *Pennisetum giganteum*, hydrogen-rich water, plant growth-promoting rhizobacteria, salinity stress, transcriptome

## Abstract

Soil salinization severely restricts agricultural production and the sustainable use of soil. While plant growth-promoting rhizobacteria (PGPR) and hydrogen-rich water (HRW) have individually been reported to alleviate salt tolerance, their synergistic effects and molecular mechanisms remain largely unexplored. In this study, we investigated the combined application of a salt-tolerant PGPR strain *Cytobacillus firmus* L71 and HRW in *Pennisetum giganteum* under NaCl stress. A factorial pot experiment was conducted under three salt levels (0, 250, and 500 mM NaCl) with or without PGPR-HRW treatment. Growth traits, antioxidant activities, osmotic regulators, and transcriptomic responses were measured. The combined treatment significantly promoted growth under severe salinity, with shoot fresh weight increasing by 148% and root length by 54.60% compared with untreated control. Physiological measurements showed elevated activities of Superoxide Dismutase (SOD), Peroxidase (POD), and Catalase (CAT), and reduced accumulation of Malondialdehyde (MDA) and Hydrogen peroxide (H_2_O_2_). Transcriptome analysis indicated consistent enrichment in plant hormone signaling, mitogen-activated protein kinase (MAPK) signaling, and plant-pathogen interaction pathways. Negative regulators such as CaM/CML (induces stomatal closure), CDPK (triggers hypersensitive response), WRKY25/33 (inhibits DNA defense genes), and JAZ (accelerates stress-induced senescence) were down-regulated, while positive regulators including A-ARR (enhances cell division and shoot growth) were up-regulated, contributing to sustained stomatal function, delayed senescence, and improved reactive oxygen species (ROS) balance. These results demonstrate that PGPR-HRW synergy enhances salt tolerance through coordinated physiological and transcriptional regulation, highlighting the potential of integrating microbial inoculants with HRW for sustainable saline soil remediation and crop improvement.

## Graphical Abstract

Physiological and molecular mechanisms underlying the induction of salt tolerance in megacephala by PGPR-HRW co-treatment. (1) Maintaining normal stomatal opening; (2) Maintaining reactive oxygen species (ROS) balance; (3) Delayed hypersensitivity (HR) response; (4) Reduces ethylene (ETH) synthesis and slows down aging; (5) Promote cytokinin (CTK) synthesis.The abstract should ideally be structured according to the IMRaD format (Introduction, Methods, Results and Discussion). Provide a structured abstract if possible. If your article has been copyedited by us, please provide the updated abstract based on this version.

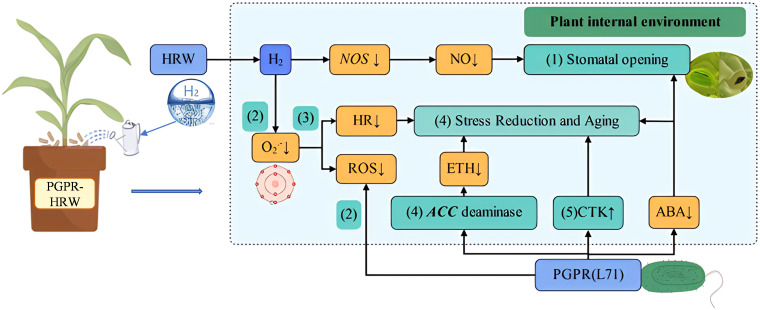

## 1 Introduction

Among various abiotic stresses, soil salinity significantly constrains crop yields and global sustainable development (Mukhopadhyay et al., 2021; Litalien and Zeeb, 2020). According to the Food and Agriculture Organization (FAO), salt-affected soils cover 424 million hectares of topsoil (0–30 cm) and 833 million hectares of subsoil (30–100 cm), based on 73% of mapped land (Negacz et al., 2022). In China, nearly 97 million hectares of saline soil are primarily distributed in the North China Plain, the Yellow River Hetao Plain, and the northwest inland area (Bian et al., 2021). The area of saline soils is increasing at a rate of 10% per year (Sharif et al., 2019). Soil salinization adversely affects plant growth by causing osmotic imbalance, which hinders the absorption of water and nutrients (Santander et al., 2020). Additionally, salt stress induces ion imbalance and oxidative stress in plants, leading to secondary stress reactions and potentially severe outcomes, including plant death (Li et al., 2017; Balasubramaniam et al., 2023).

Phytoremediation using halophytic or salt-tolerant plants for saline soil remediation is low-cost, sustainable, and ecologically beneficial. It is an effective measure for efficient saline soil utilization (Srivastava, 2020). *Pennisetum giganteum* Z.X. Lin, a perennial C4 grass species in Poaceae family, was characterized by its rapid growth, extensive root system, and strong tillering ability (Hayat et al., 2022). In recent years, it had been gradually applied in the remediation of heavy metal-contaminated soils (Yankey et al., 2021) and in the improvement of saline soils. Previous research had shown that *P. giganteum* exhibited good salt tolerance, being able to withstand light to moderate salt stress without affecting its biomass (Hayat et al., 2020).

To achieve better plant improvement efficiency, various remediation technologies were applied to saline soil improvement, with PGPR being one of them. PGPRs are soil bacteria that alleviate the adverse effects of ethylene on plants by producing ACC deaminase, which decomposes the ethylene precursor (Gupta et al., 2022). They accelerated phosphate solubilization, produced extracellular polysaccharide, iron-producing carriers, and volatile compounds (Kumawat et al., 2022; Ha-Tran et al., 2021), and synthesized various plant hormones such as auxins (IAA), cytokinins (CTK), and gibberellins (GA) (Saleem et al., 2021; Li et al., 2020). Additionally, they maintained normal plant growth under salt stress through multiple pathways, including increasing the potassium to sodium ion ratio, reducing intracellular electrolyte leakage, and promoting nutrient and water uptake (Bhat et al., 2020).

On the other hand, H_2_, as a novel gaseous signaling molecule, was shown to have promising applications in enhancing plant salt tolerance. When H_2_ was supplied to plants as HRW, it acted as a beneficial gaseous molecule in adaptive responses. HRW enhanced plant stress resistance by improving antioxidant system activity and osmotic regulation capacity (Hu et al., 2021). The molecular mechanisms by which H_2_ promoted plant stress resistance involved the regulation of miRNA, gene expression, hormone levels, and protein modifications, and were possibly related to various gaseous signaling pathways such as Nitric Oxide (NO) and carbon monoxide (CO) (Wang et al., 2022; Chen et al., 2017). Existing studies indicated that HRW showed good effects in alleviating various abiotic stresses such as drought, salinity, heavy metal stress, and extreme temperatures in plants (Wang et al., 2023). Under salt stress, HRW enhanced plant salt tolerance by re-establishing reactive oxygen species homeostasis and ion homeostasis (Su et al., 2021). However, previous studies mainly focused on single remediation methods, the effects and molecular mechanisms of combined PGPR-HRW remediation remained unclear.

In this study, we conducted a factorial experiment in *P. giganteum* that combined NaCl salinity with a treatment using a salt-tolerant PGPR and HRW. The objectives were to: (1) quantify the effects of the PGPR-HRW treatment on seedling growth and biomass across different NaCl levels; (2) determine changes osmotic adjustment, oxidative status, and antioxidant enzyme activities under PGPR-HRW treatment; and (3) investigate the molecular mechanism for alleviating salt stress under PGPR-HRW treatment. These analyses provide a physiological and molecular basis for the alleviation of salt stress by PGPR-HRW and offer guidance for its application in the remediation of salinized soil.

## 2 Materials and methods

### 2.1 Plant materials, growth conditions, and stress treatment

In our previous research, a salt-tolerant growth-promoting bacterium, *C.firmus* L71, was isolated from saline-alkaline coastal soil (16S rDNA gene sequence registration number in GenBank: OP935756. CGMCC depository number: 26877). Pot experiments with *P. giganteum* under different salt stress were conducted with varying concentrations of HRW and L71 strain. It was found that combined treatment of L71 strain and 50% HRW had the best growth-promoting effect on *P. giganteum* (**Supplementary Figures S1**-**S3**). Principal component analysis (PCA) results showed that the 50% HRW-L71 combined treatment effectively alleviated the effects of 250 mM and 500 mM salt stress on *P. giganteum*. The highest overall score at the same concentration was obtained under both salt stress conditions (**Supplementary Figure S4**). Therefore, the 50% HRW-L71 combined treatment was selected for further experiments as the subsequent PGPR-HRW treatment combination.

The effect of PGPR-HRW was studied through a two-factor pot experiment. Three levels of NaCl stress were applied: 0 mM (A0), 250 mM (A1), and 500 mM (A2). For each salt stress, two treatments were applied: without PGPR-HRW (B0) and with PGPR-HRW (B1). This resulted in six treatment groups (A0B0, A0B1, A1B0, A1B1, A2B0, A2B1), each with ten replicates. Conducted at Shanghai Jiao Tong University (31°11′N, 121°36′E). After 14 days of planting, NaCl treatment and PGPR-HRW treatment were applied. Salt treatment was conducted every 20 days at a volume of 100 ml per pot. L71 was dissolved in hydrogen-rich water as a liquid inoculant and applied via irrigation at 100 ml per pot per application. The detailed procedure involved centrifuging the fermented culture at 4 °C and 12,000 rpm for 10 minutes. After decanting the supernatant, the resulting bacterial pellet was uniformly dissolved in hydrogen-rich water to prepare a liquid inoculum solution with a concentration of 10^8^ CFU/mL. 50% HRW (0.8 ppm H_2_) was prepared using CA/H-1 hydrogen generator. Control groups received the same volume of water. After 40 days, *P. giganteum* was harvested for indicator measurements.

### 2.2 Determination of plant morphological and physiological indicators

After 40 days of treatment, phenotypic traits were determined by measuring plant height, root length, fresh and dry weights of shoot and root parts (Hayat et al., 2020). The activities of SOD, POD, and CAT, as well as the contents of H_2_O_2_, MDA, proline (Pro), and soluble sugars (SS), were measured using kits provided by the Nanjing Jiancheng Bioengineering Institute.

### 2.3 Transcriptomic analysis

Plant leaves subjected to different treatments for 40 d were sampled, and their leaves were rinsed thrice with ultrapure water and immediately frozen in liquid nitrogen. Total RNA from *P. giganteum* leaves was extracted using TRIzol^®^ Reagent. The specific methods and quality requirements for RNA extraction were described in **Supplementary Text S1**. RNA purification, reverse transcription, library construction, and sequencing were performed at Shanghai Majorbio Bio-pharm Biotechnology Co., Ltd. (Shanghai, China) according to the manufacturer’s instructions. Specific methods, processes, and sequencing platform were described in **Supplementary Text S2**. RNA-seq was performed on four treatment combinations (A0B0, A0B1, A2B0, A2B1), each with three biological replicates (total n = 12). Sequencing generated 88.64 Gb of clean reads in total (≥5.98 Gb per sample; Q30 ≥95.92%; **Supplementary Table S1**). Clean reads from all samples were assembled *de novo* with Trinity, yielding 108308 unigenes and 210290 transcripts (mean length: 955.7 bp; N50: 1689 bp). Reads were mapped back to the assembly with rates of 85.01-86.03% (**Supplementary Table S2**). Detailed processes for quality control and *de novo* assembly were described in **Supplementary Text S3**. The methods and parameter settings for differential expression analysis and Kyoto Encyclopedia of Genes and Genomes (KEGG) functional enrichment analysis were described in **Supplementary Text S4**. Read counts were normalized with DESeq2 using the median-of-ratios procedure. Differential expression was called at |log_2_FC| ≥ 1 with FDR < 0.05.

### 2.4 Quantitative real-time PCR validation

12 candidate DEGs revealed by transcriptome sequencing were randomly selected for further validation of differential expression by qRT-PCR. The DEGs ID, annotation description (NR) and primer pairs used were listed in **Supplementary Table S3**. Briefly, total RNA was extracted using the RNAprep Pure Plant Kit (TIANGEN, DP432). Total RNA from each sample was then reverse transcribed using reverse transcriptase MMLV (TaKaRa, RR047A) and used as a template. qRT-PCR was performed with TB Green^®^ Premix Ex Taq™ II (TaKaRa, RR820A) on an ABI 7500 System (Applied Biosystems, Foster City, CA) (Gutsch et al., 2019). GAPDH was used as an internal control to normalize gene expression levels. The relative expression of each DEG was evaluated using the 2^-ΔΔCt^ method (Zhao et al., 2021). For each gene and sample, 3 biological replicates and 3 technical replicates were used.

### 2.5 Statistical analysis

The RNA-Seq assay was performed in triplicate for each treatment. Bar charts were generated using Origin 2024 software. Gene expression heatmaps were created using R 4.3.2. Analysis of variance (ANOVA) was conducted for statistical analysis with SPSS 27 software, followed by multiple pairwise comparisons using Duncan’s test at the *p* < 0.05 level. Morphological and physiological indices were determined using 4 replicates. The two-way ANOVAs of SAS software (SAS Institute, Cary, NC) was used to indicate the effect of NaCl and PGPR-HRW treatments on the test variables.

## 3 Results

### 3.1 PGPR-HRW combined treatment promoted the growth of *P. giganteum* under high salt stress

**Figure 1:**
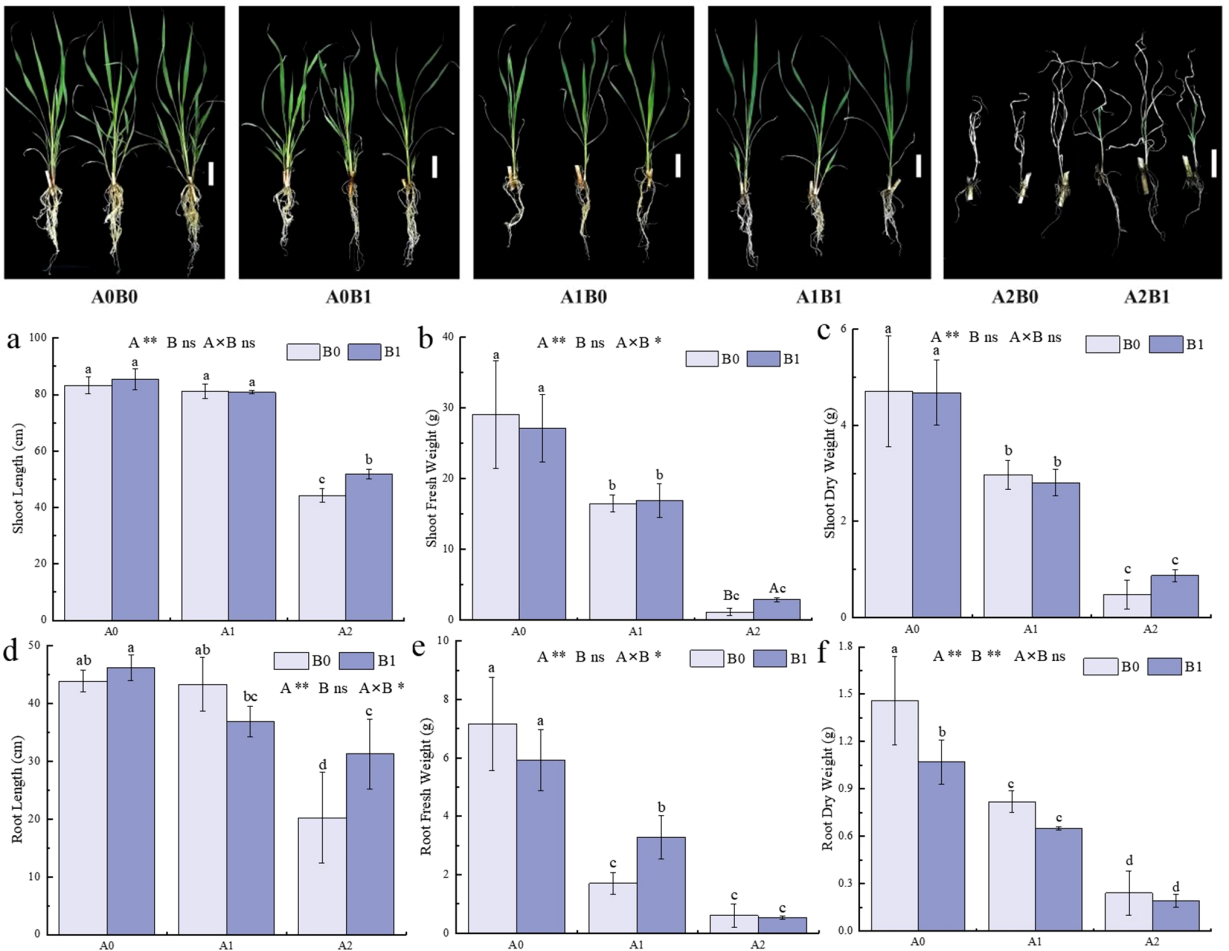
Phenotype and Growth characteristics of *P. giganteum* between all treatments. A0: 0mM NaCl, A1:250mM NaCl, A2:500mM NaCl. B0: no PGPR-HRW treatment, B1: PGPR-HRW treatment. Scale bar = 10 cm. **(a-c)** Shoot length **(a)**, fresh weight **(b)** and dry weight **(c)**. **(d-f)** Root length **(d)**, fresh weight **(e)** and dry weight **(f)**. Different lowercase letters indicate significant differences between treatments (*p*<0.05), different capital letters indicate significant differences between the two groups under the same concentration of salt treatment (*p*<0.05). The results of two-way ANOVA are listed as: A, NaCl effect; B, PGPR-HRW effect; A×B, interaction effect; *p < 0.05; **p < 0.01; ns, not significant.

The growth of *P. giganteum* under 40 days of different salt stress was shown in **Figure 1**. The combined treatment of salt stress and PGPR-HRW significantly affected both shoot fresh weight, the root length and root fresh weight (*p* < 0.5). As the salt stress increased, tillering of *P. giganteum* gradually decreased, and the leaves turned yellow. Under treatment A2, the degree of leaf yellowing was lower in treatment B1 compared to treatment B0. The impact of the PGPR-HRW combined treatment on the growth of *P. giganteum* was further compared under different salt stress. Under no salt stress (A0), the difference between B1 and B0 treatments was minimal. Under 250 mM (A1) salt stress, the root fresh weight in the B1 treatment was significantly higher than in the B0 treatment, increasing by 92.94% (**Figure 1e**). At a salt stress of 500 mM (A2), the growth advantage under the B1 treatment became more apparent. Plant height, root length, and shoot fresh weight in A2B1 treatment were significantly higher than A2B0 treatment, increasing by 17.13%, 54.60%, and 148%, respectively. The shoot dry weight increased by 81.25% (**Figures 1a, b, d**). Additionally, it was observed that there was no significant difference in plant height and root length between the A1B0 and A0B0 groups (**Figures 1a, d**), indicating that *P. giganteum* has certain level of salt tolerance. The PGPR-HRW combined treatment exhibited better growth-promoting effects under high salt stress.

### 3.2 PGPR-HRW combined treatment enhanced the physiological activity of *P. giganteum* under salt stress

**Figure 2:**
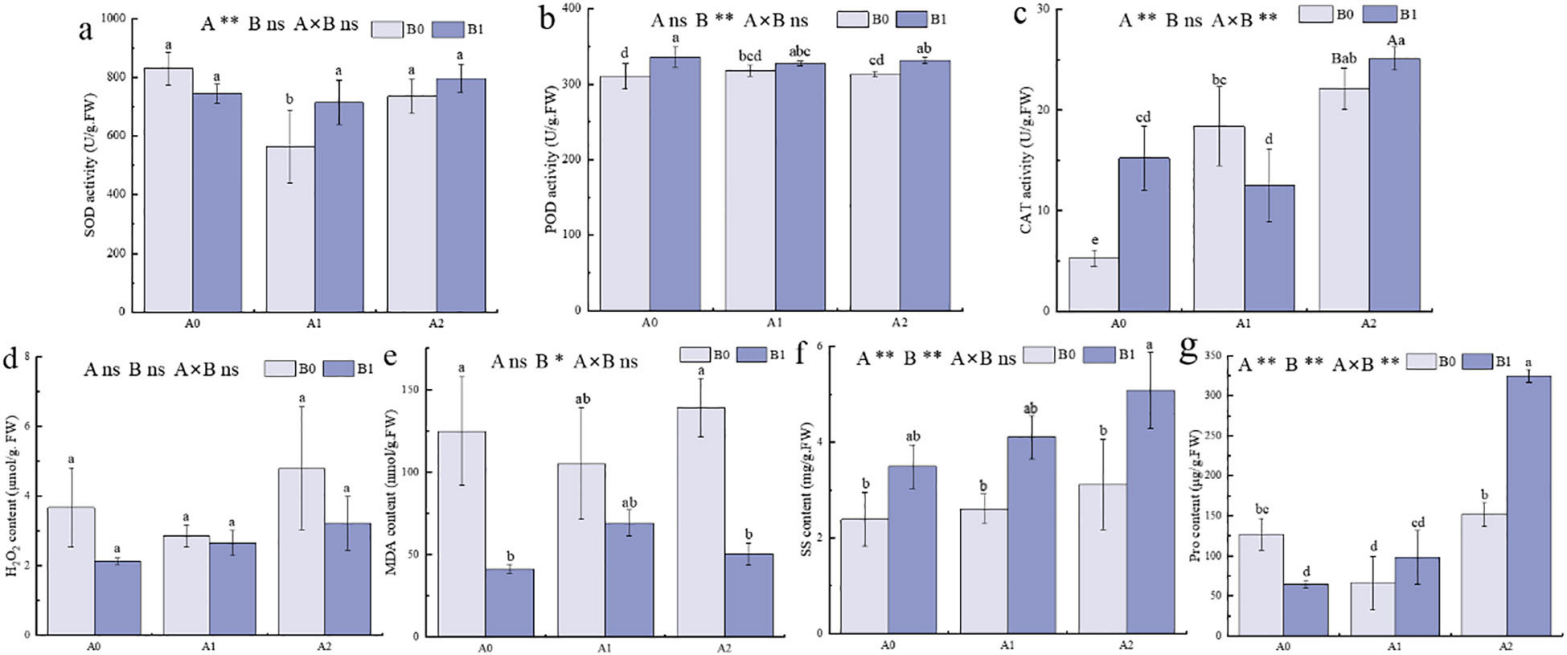
Physiological characteristics of *P. giganteum* between all treatments. A0: 0mM NaCl, A1:250mM NaCl, A2:500mM NaCl. B0: no PGPR-HRW treatment, B1: PGPR-HRW treatment. **(a–c)** SOD **(a)**, POD **(b)** and CAT **(c)** activities. **(d–f)** H_2_O_2_ **(d)**, MDA **(e)**, SS **(f)** and Pro **(g)** contents. Different lowercase letters indicate significant differences between treatments (*p*<0.05), different capital letters indicate significant differences between the two groups under the same concentration of salt treatment (*p*<0.05). The results of two-way ANOVA are listed as: A, NaCl effect; B, PGPR-HRW effect; A×B, interaction effect; *p < 0.05; **p < 0.01; ns, not significant.

Salt stress disrupted the ROS scavenging balance, leading to oxidative stress (Yan et al., 2016). Therefore, physiological measurements of *P. giganteum* under salt stress were performed. The results indicated that under no salt stress (A0), the B1 treatment increased POD and CAT activities and decreased MDA content in *P. giganteum* (**Figures 2b, c, e**). When salt stress increased to 250 mM (A1), SOD activity in the B1 treatment was significantly higher than in the B0 treatment by 26.63%, and soluble sugar content increased by 57.36% (**Figures 2a, f**). When salt stress continued to increase to 500 mM (A2), POD and CAT activities in the B1 treatment were higher than B0 treatment by 5.99% and 13.48%, respectively (**Figures 2b, c**). The contents of SS and Pro increased significantly by 63.21% and 114%, respectively (**Figures 2f, g**), while MDA content decreased significantly by 63.89% and H_2_O_2_ content dropped by 48.78% (**Figures 2d, e**). This indicated that with increasing salt stress, the PGPR-HRW combined treatment enhanced the antioxidant enzyme activity of *P. giganteum*, maintained osmotic regulation balance, and reduced the production of peroxidation products such as MDA and H_2_O_2_. The combined treatment of salt stress and PGPR-HRW had a highly significant effect on both CAT and proline (*p* < 0.01).

### 3.3 Global analysis of differentially expressed genes

**Figure 3:**
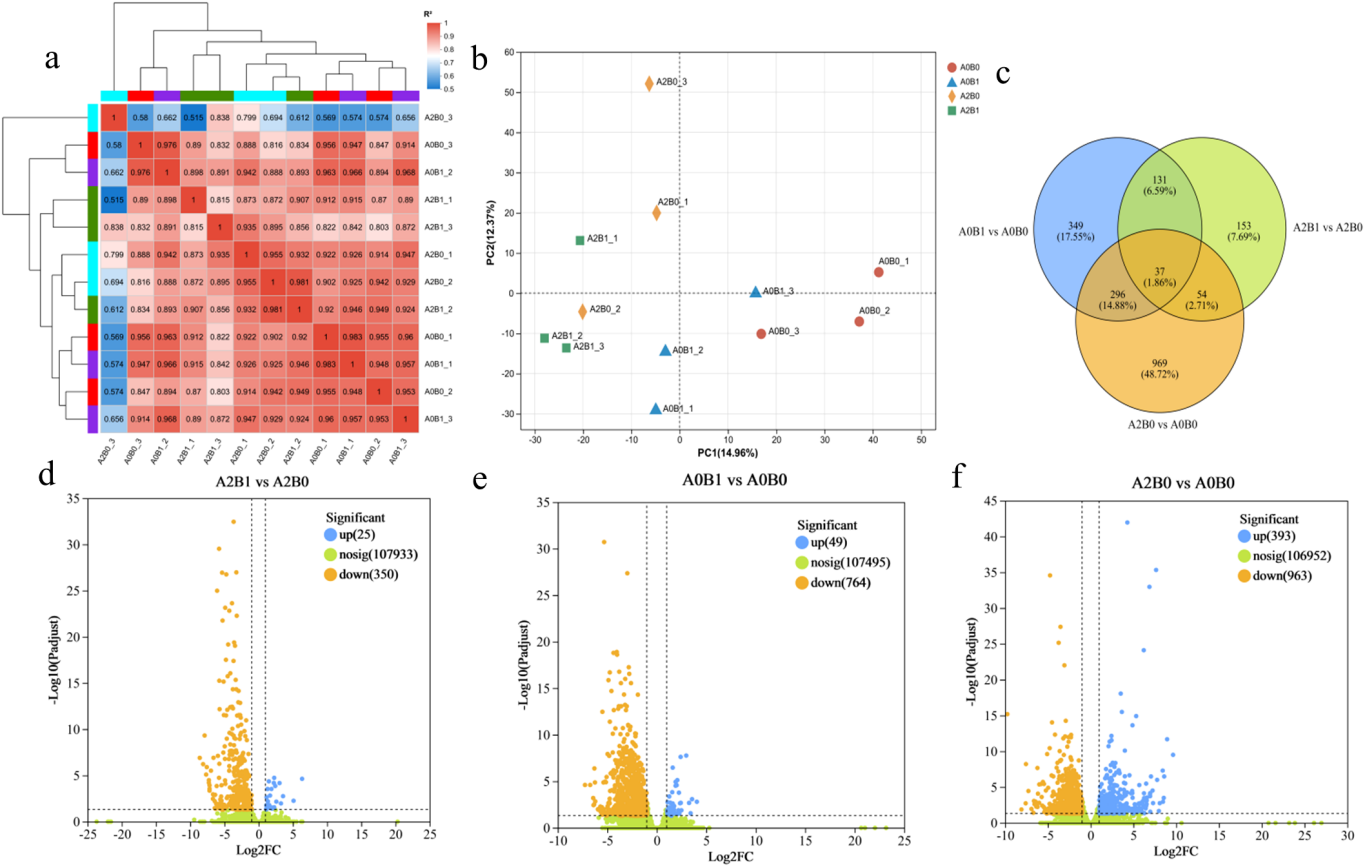
Overview analysis of up-regulated and down-regulated genes (DEGs) in different comparison groups. **(a)** Correlation studies among all treatments, including A0B0, A0B1, A2B0 and A2B1. **(b)** PCA analysis. **(c)** Venn diagram representing the unique and common DEGs. **(d–f)** Numbers of up and down-regulated genes in all comparison groups: A2B1 vs A2B0, A0B1 vs A0B0, A2B0 vs A0B0.

Biological replicates showed high pairwise correlation (R^2^ between 0.8 and 1), supporting data reliability (**Figure 3a**). PCA analysis showed distinct gene cluster expression patterns across the different treatments (**Figure 3b**). The differences in DEGs among three comparison groups were analyzed: A2B1 vs A2B0, A0B1 vs A0B0, and A2B0 vs A0B0. In the comparison of A2B1 vs A2B0, a total of 375 DEGs were identified, with 25 genes upregulated and 350 genes downregulated (**Figure 3d**). For A0B1 vs A0B0, 813 DEGs were identified, with 49 genes upregulated and 764 genes downregulated (**Figure 3e**). In the comparison of A2B0 vs A0B0, 1356 DEGs were identified, with 393 genes upregulated and 963 genes downregulated (**Figure 3f**). The unique DEGs for A2B1 vs A2B0, A0B1 vs A0B0, and A2B0 vs A0B0 were 153, 349, and 969 respectively. There were 168 common DEGs between A2B1 vs A2B0 and A0B1 vs A0B0; 91 common DEGs between A2B1 vs A2B0 and A2B0 vs A0B0; and 33 common DEGs between A0B1 vs A0B0 and A2B0 vs A0B0. Among these, 37 DEGs were common across all three comparisons (**Figure 3c**).

**Figure 4:**
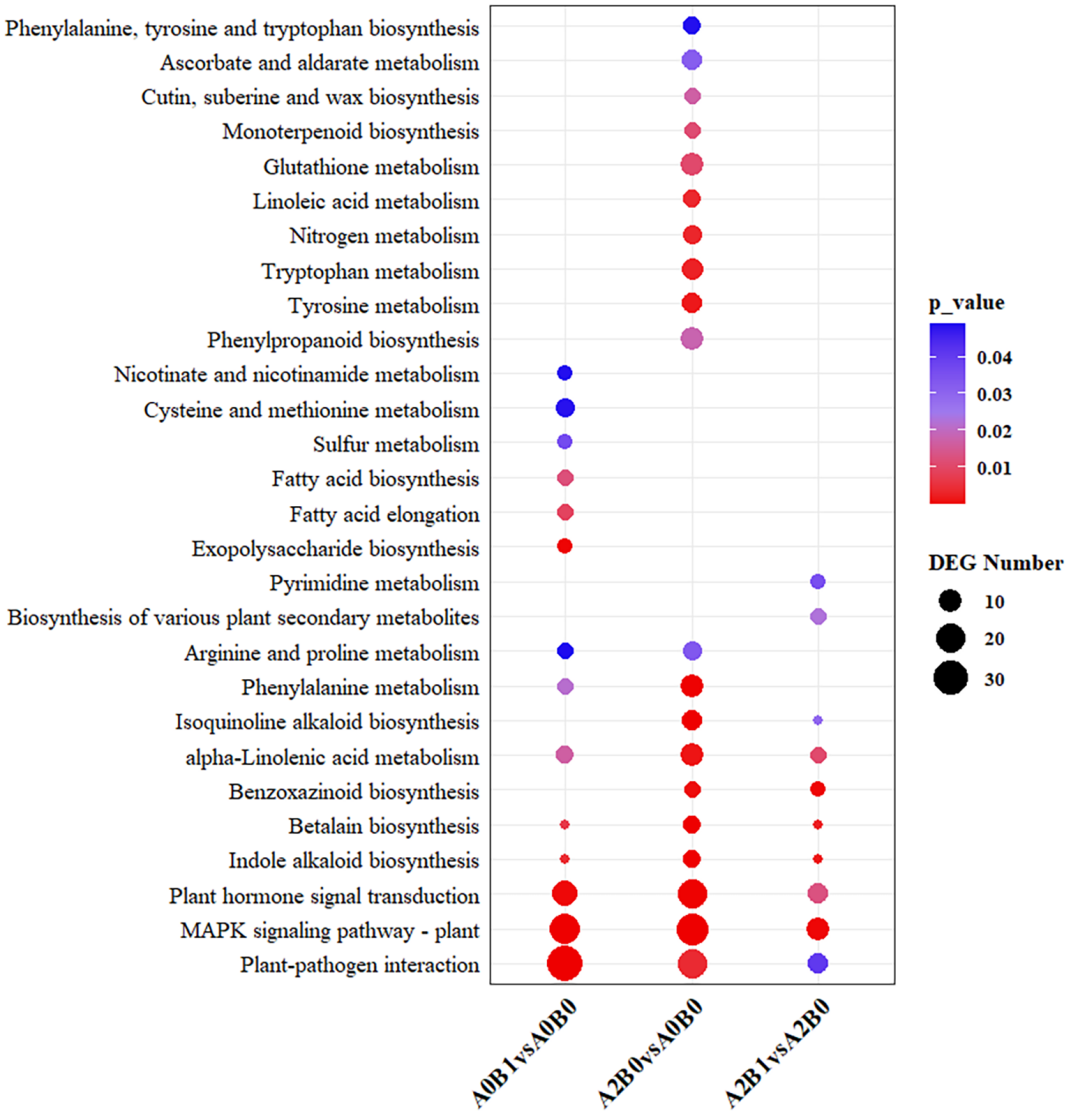
The significantly (*p*<0.05) enriched pathways for DEGs of A2B1 vs A2B0, A0B1 vs A0B0, A2B0 vs A0B0 in KEGG analysis.

KEGG pathway analysis was conducted to explore the biological pathways represented by DEGs in the three comparison groups. There were 10, 14 and 20 enriched pathways in the comparisons: A2B1 vs A2B0, A0B1 vs A0B0, A2B0 vs A0B0 (*p* < 0.05). The “MAPK signaling pathway – plant”, “plant-pathogen interaction”, and “plant hormone signal transduction” pathways were significantly enriched and contained the highest number of differentially expressed genes (DEGs) across all three comparison groups (**Figure 4**). These three pathways were thus identified as the primary common pathways. In addition, the “Indole alkaloid biosynthesis”, “Betalain biosynthesis”, “Phenylalanine metabolism”, and “Arginine and proline metabolism” pathways were consistently enriched in all three comparison groups. Specifically, the “Biosynthesis of various plant secondary metabolites” and “Pyrimidine metabolism” pathways were enriched only in the A2B1 vs A2B0 comparison. The “Exopolysaccharide biosynthesis”, “Fatty acid elongation”, and “Fatty acid biosynthesis” pathways were specific to A0B1 vs A0B0. Meanwhile, the “Phenylpropanoid biosynthesis”, “Tyrosine metabolism”, and “Tryptophan metabolism” pathways were uniquely enriched in A2B0 vs A0B0. This suggested that the B1 (PGPR-HRW) treatment may have a considerable overlap with the inherent salt tolerance mechanisms of *P. giganteum*, and B1 treatment could enhance certain inherent salt tolerance pathways of *P. giganteum*.

### 3.4 Analysis of “plant hormone signal transduction pathway”, “plant-pathogen interaction” and “MAPK signaling pathway – plant”

#### 3.4.1 Plant hormone signal transduction pathway

KEGG analysis showed significant enrichment of “Plant hormone signal transduction pathway”, “Plant-pathogen interaction and “MAPK signaling pathway – plant” in all three comparison groups (**Figure 4**). Key regulatory nodes and expression patterns within these pathways were further examined.

**Figure 5:**
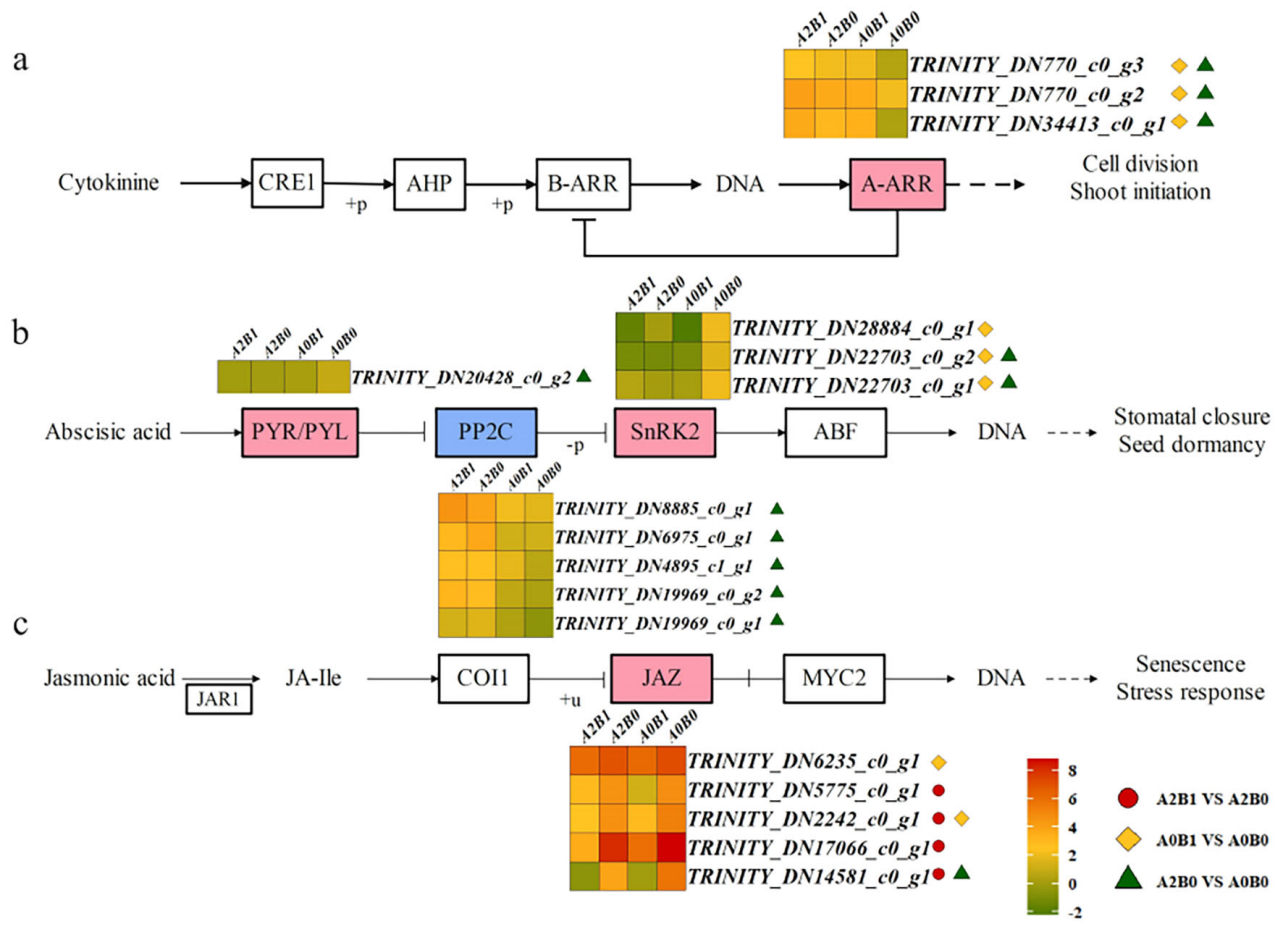
Different contrast groups activated ‘hormone signal transduction’ pathway. **(a)** CTK pathway; **(b)** ABA signaling pathway; **(c)** JA signaling pathway. 2.Heat maps showing the log2 expression values of DEGs in each treatment. 3.The color ‘
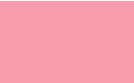
’ indicates a positive regulator of the final expression function, and the color ‘
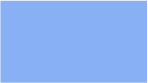
’ indicates a negative regulator of the final expression function. 4.Three different marks indicate that the difference in the expression of DEGs in the corresponding comparison groups reached a significant level. Notes 2–4 below.

In the plant hormone signal transduction pathway, DEGs were enriched in pathways mediated by CTK, abscisic acid (ABA), and jasmonic acid (JA). In the CTK pathway, CTK bound to the receptor CRE1, inducing phosphorylation of AHP, which translocated to the nucleus and phosphorylated B-ARR, regulating A-ARR to control cell division and bud sprouting (**Figure 5a**). High expression of 3 ARR-A DEGs was observed in all comparison groups, with significant differences in A0B1 vs A0B0 and A2B0 vs A0B0 (**Figure 5a**). These results suggested that B1 treatment enhanced cell division and bud sprouting in *P. giganteum* under salt stress.

In the ABA signaling pathway, the intracellular receptor PYR/PYL inhibited PP2C (protein phosphatase 2C), releasing SnRK2 (serine/threonine-protein kinase SRK2). Activated SnRK2 regulated downstream pathways controlling stomatal closure and seed dormancy (**Figure 5b**). A total of 1, 5, and 3 DEGs were identified for PYR/PYL, PP2C, and SnRK2, respectively (**Figure 5b**). In the A2B0 vs A0B0 group, 5 PP2C DEGs were significantly up-regulated, whereas 3 SnRK2 DEGs were down-regulated in A0B1 vs A0B0. Elevated PP2C and reduced SnRK2 expression suppressed stomatal closure, cooperating with the CaM/CML pathway to maintain normal stomatal opening in *P. giganteum*.

In JA signaling pathway, JA was catalyzed by jasmonate amino acid synthase JAR1 to form JA-lle (jasmonoyl-isoleucine). JA-lle acted on COI1, entered nucleus and inhibited the dissociation of JAZ from MYC2 after ubiquitination, thereby regulating senescence and stress responses (**Figure 5c**). 5 JAZ DEGs were differentially expressed and consistently down-regulated across all comparison groups, with four showing significant down-regulation in A2B1 vs A2B0 (**Figure 5c**). This indicated that under A2 salt stress, the B1 treatment could inhibit plant senescence and stress responses, supporting normal plant growth.

#### 3.4.2 Plant-pathogen interaction pathway

**Figure 6:**
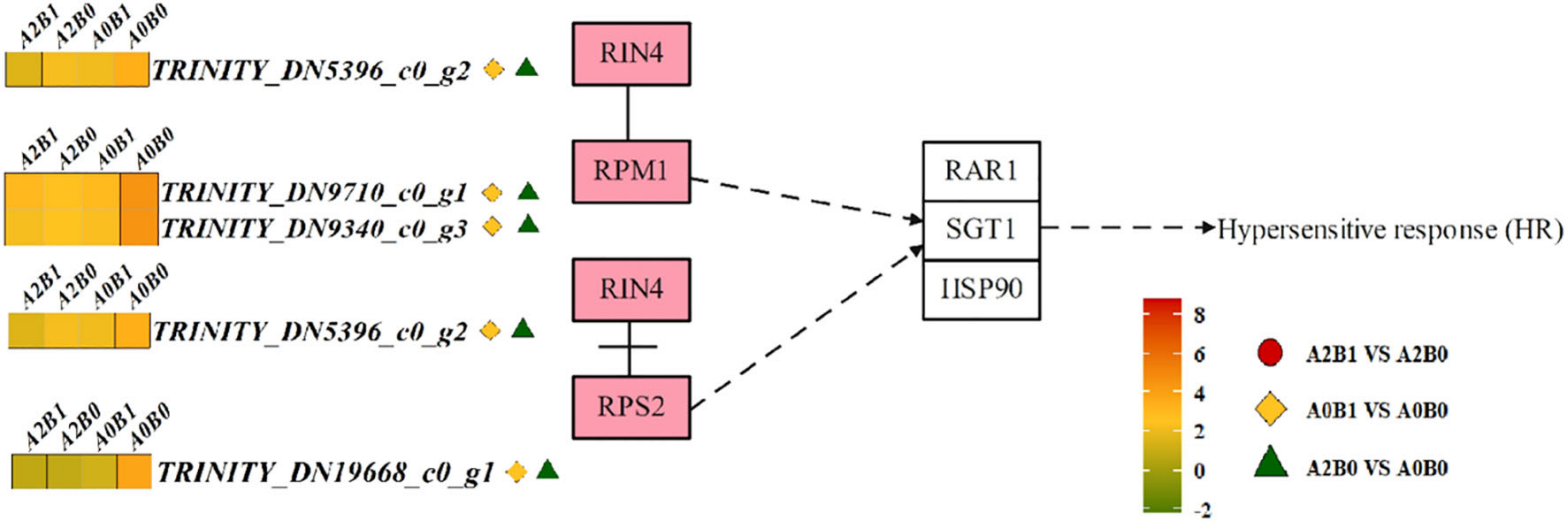
Different contrast groups activated ‘Plant-pathogen interaction pathway’ pathway. RPM1/2 disease resistance proteins initiate HR signaling pathway.

In the Plant-pathogen interaction pathway, cytoplasmic RPM1/2-type resistance proteins (RIN4, RPM1, RPS2) mediated hypersensitive response (HR). The RIN4 protein dissociated from RPS2 and associated with RPM1, indirectly inducing the expression of RAR1, suppressor SGT1, and heat shock protein HSP90, thereby triggering intracellular HR (**Figure 6**). 1 *RIN4* DEG, 2 *RPM1* DEGs, and 1 *RPS2* DEG were down-regulated in A0B1 vs A0B0 and A2B0 vs A0B0. In A2B1 vs A2B0, except for 1 DEG of *RPM1* (TRINITY_DN9710_c0_g1) was up-regulated, the other 3 DEGs also showed down-regulated expression trend (**Figure 6**). The down-regulation of these genes could indirectly inhibit the HR response, reducing browning and senescence of plant leaves.

#### 3.4.3 Plant-pathogen and MAPK signaling-plant interactions pathway

**Figure 7:**
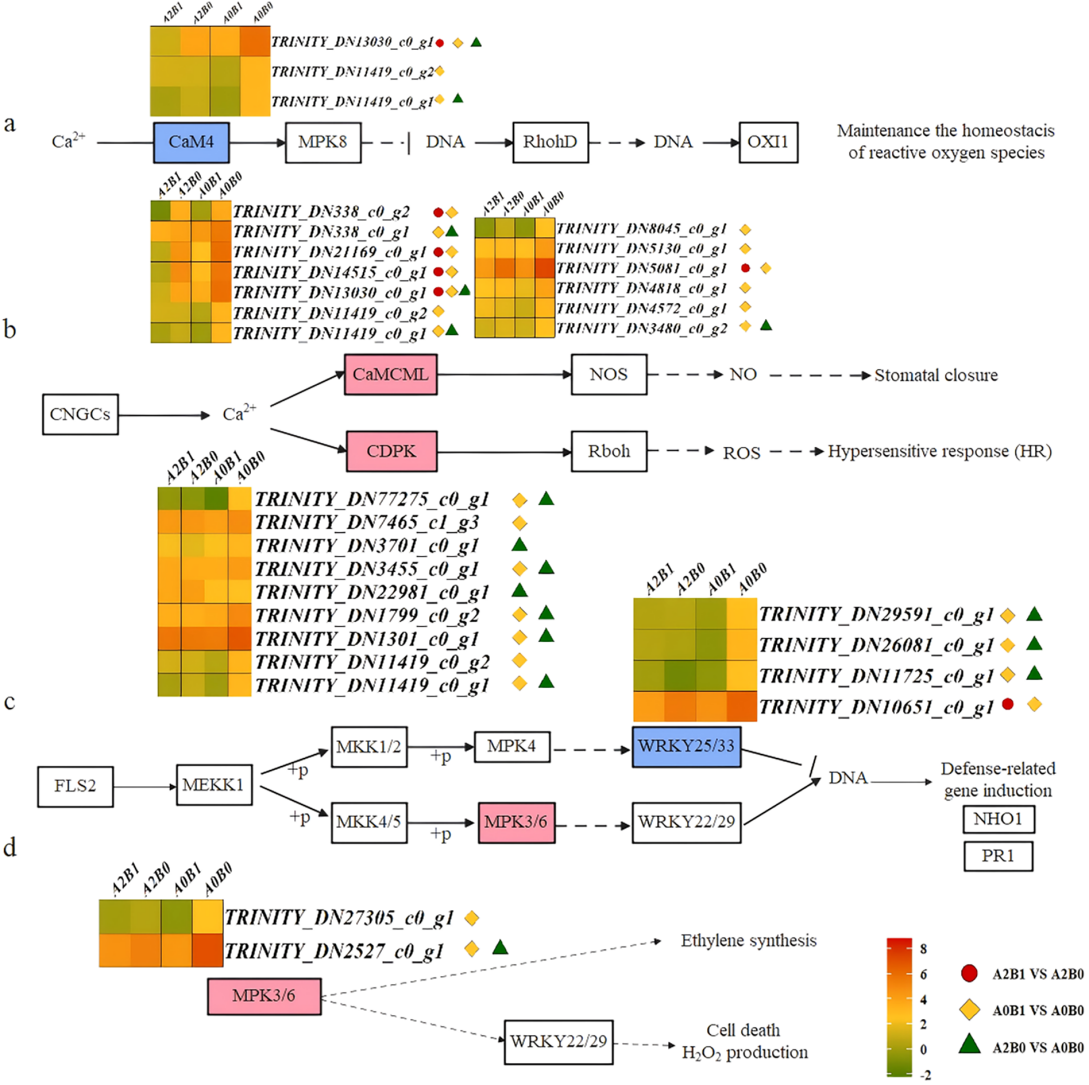
Different contrast groups activated “Plant-pathogen interaction pathway” and “MAPK signaling pathway – plant”. **(a)** “MAPK signaling pathway – plant” Ca^2+^-CaM4 dependent signal pathway, **(b)** “Plant-pathogen interaction” Ca^2+^-dependent signal pathway, **(c)** “Plant-pathogen interaction” LRR receptor-like serine/threonine protein kinase (FLS2) initiated mitogen-activated protein kinase signaling pathway, **(d)** “MAPK signaling pathway – plant” MPK3/6 signal pathway.

In the Ca^2+^-CaM4 dependent signaling pathway of the MAPK signaling pathway - plant, Ca^2+^ signals were transduced by CaM4, which suppressed the expression of OXI1, a serine/threonine kinase transcription factor maintaining ROS balance. Separate comparisons revealed that 1 CaM4 DEGs was significantly down-regulated between B0 and B1 at A2, 3 CaM4 DEGs were down-regulated between B0 and B1 at A1, and 2 CaM4 DEGs were down-regulated between A0 and A2 at B0. TRINITY_DN13030_c0_g1 was commonly down-regulated in all these comparisons (**Figure 7a**). CaM4 activated MPK8, negatively regulating RbohD to maintain ROS homeostasis. This indicated that under salt stress, *P. giganteum* down-regulated CaM4 to balance ROS, and B1 treatment enhanced this effect. Notably, 3 DEGs (TRINITY_DN13030_c0_g1, TRINITY_DN11419_c0_g1, and TRINITY_DN11419_c0_g2) also participated in the Ca^2+^-dependent signaling pathway of plant-pathogen interactions, functioning in CaM/CML (calmodulin/calcium-binding proteins). Here, Ca^2+^ signals were transduced by CaM/CML or CDPK, activating stomatal closure and ROS-dependent hypersensitive response (HR). CaM/CML regulated NOS (nitric oxide synthase) activity, influencing NO production and inducing stomatal closure and HR. Among 13 CaM/CML DEGs, all were significantly down-regulated under B1 treatment compared to B0, and down-regulated in A2B0 vs A0B0 (**Figure 7b**). This repression likely helped maintain stomatal opening under salt stress, an effect intensified by B1. Similarly, in the expression of CDPK calcium-dependent protein kinases, with the exception ofTRINITY_DN22981_c0_g1 was up-regulated in A2B0 vs A0B0, the other 8 DEGs were significantly repressed in A0B1 vs A0B0 or A2B0 vs A0B0 (**Figure 7b**). CDPK promoted the phosphorylation of Rboh (respiratory burst oxidase), activating the ROS-dependent HR in plants. The down-regulation of CDPK related gene expression inhibited this process.

Another Plant-pathogen interaction pathway was initiated by the LRR receptor-like serine/threonine kinase FLS2, activating MAPK signaling. This defense response was mediated by MEKK1-MKK1/2-MPK4-WRKY25/33 or MEKK1-MKK4/5-MPK3/6-WRKY22/29 cascades. In these pathways, 4 *WRKY25/33* DEGs were specifically expressed across three comparison groups (**Figure 7c**). Both B1 and A2 treatments significantly suppressed *WRKY25/33* expression. As negative transcriptional regulators, WRKY25/33 inhibited defense-related genes like *NHO1* and *PR1*. Their down-regulation enhanced defense gene expression via a double-negative mechanism, promoting salt stress defense in *P. giganteum*. Additionally, MPK3/6, involved in both pathways, acted as ethylene (ETH) synthesis inducers, regulating H_2_O_2_ production and cell death. MPK3/6 showed down-regulation in all comparison groups (**Figure 7d**), suggesting that B1 treatment delayed leaf senescence by suppressing ETH synthesis.

### 3.5 Validation of the DEGs results by qRT-PCR analysis

**Figure 8:**
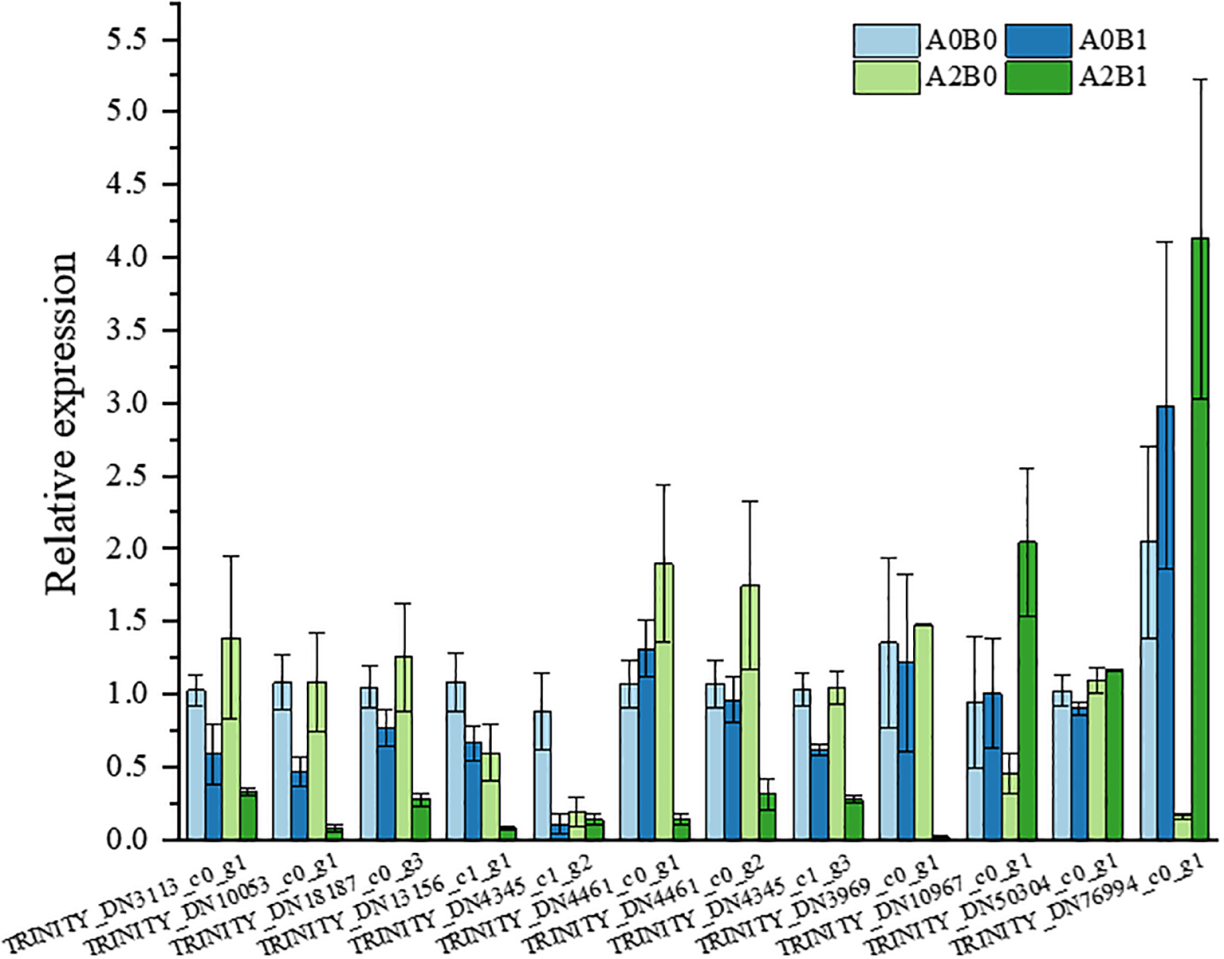
Validation of DEGs expression by qRT-PCR analysis of randomly selected genes.

To validate the reliability of RNA-Seq data, 12 DEGs associated with ethylene activation signaling, *NAC* domain-containing proteins, and glutathione transferase activity were selected and tested by qRT-PCR (**Supplementary Table S3**). The relative expression trends of selected DEGs were similar to those observed in RNA-Seq data and were consistent with Illumina sequencing results (**Figure 8**), demonstrating the reliability of RNA-Seq data.

## 4 Discussion

### 4.1 Effects of PGPR-HRW combined treatment on the growth and physiology of *P. giganteum* under salt stress

The combined application of PGPR and HRW markedly improved the growth and salt tolerance of *P. giganteum*. The results showed that the plant height, root length, and shoot fresh weight of *P. giganteum* treated with PGPR-HRW were significantly higher than those of the control. Moreover, the differences became more pronounced with increasing salt stress (**Figure 1**).

The physiological basis for enhanced plant growth was supported by the combined effects of the PGPR-HRW treatment on the antioxidant system and osmotic homeostasis of plant. The PGPR-HRW treatment increased activities of antioxidant enzymes (POD and CAT), and contents of Pro and soluble sugars, while reducing levels of peroxidation products (MDA and H_2_O_2_) (**Figure 2**). This finding was consistent with previous studies. The addition of NaCl was found by Wu to significantly inhibit the elongation of barley roots and cause a loss of cell viability, however, these adverse effects were significantly reversed by HRW treatment (Wu et al., 2020). Kumar isolated *Bacillus pumilus* strain JPVS11 from saline soil and inoculated it into salt-stressed rice, finding significant increases in plant height, root length, fresh and dry weight, and the activities of CAT and SOD after treatment (Kumar et al., 2021). Patani used 5 PGPR *Bacillus* strains to inoculate salt-stressed tomatoes, finding significant improvements in phenotype, antioxidant enzyme activity, and nutrient contents (magnesium, calcium, and potassium) (Patani et al., 2023). Our findings extend these reports by demonstrating that combining HRW with PGPR enhances growth in *P. giganteum* more effectively than either treatment alone, suggesting a synergistic rather than merely additive effect.

### 4.2 PGPR-HRW treatment might alleviate salt stress effects in *P. giganteum* by inhibiting negative regulatory factors

Transcriptomic analysis provided more effective means to further reveal the salt tolerance mechanisms in plants. In DEG analysis, it was found that both PGPR-HRW treatment and 500 mM salt stress treatment resulted in more down-regulated genes than up-regulated genes in *P. giganteum* (**Figures 3d–f**). The down-regulation of gene expression was observed in A2B0 vs A0B0, which is similar to the general trend of gene suppression in olive roots treated with NaCl as reported by Skodra (Skodra et al., 2023). This suggests that *P. giganteum* alleviated salt stress effects not primarily through massive activation of stress-responsive genes, but via a targeted suppression of negative regulatory factors.

KEGG pathway enrichment analysis confirmed that this repression strategy operates within key signaling pathways known to mediate stress responses, including “MAPK signaling pathway – plant”, “plant hormone signal transduction”, and “plant-pathogen interaction”. The enrichment of these pathways is a common hallmark of plant salt stress (Ma et al., 2022; Kumar et al., 2020). Mohamed’s transcriptomic analysis of rapeseed during germination under salt stress found significant enrichment in pathways related to Plant hormone signal transduction, MAPK signaling pathway-plant, and Glycolysis/gluconeogenesis (Mohamed et al., 2022). When plants under salt stress were inoculated with PGPR, they produced large amounts of IAA, ABA, or other growth factors. These regulated gene expression and metabolism, accumulated osmolytes (Pro and betaine), and increased antioxidant enzymes and their activities to enhance stress tolerance (Zahra et al., 2023; Çam et al., 2023). The Plant-pathogen interaction pathway is particularly important in the interaction between PGPR and plants and is often observed to be enriched in this pathway (Zarattini et al., 2021; Khoso et al., 2024). Li through KEGG enrichment analysis found that DEGs of *Nitraria sibirica Pall* under salt stress mainly included Plant-pathogen interaction, Plant hormone signal transduction, and β-alanine metabolism (Li et al., 2021). Our KEGG pathway enrichment analysis results were highly consistent with previous studies. Importantly, the overlap between PGPR-HRW induced changes and the plant’s inherent salt tolerance pathways suggests that the combined treatment reinforces intrinsic mechanisms rather than activating entirely novel processes.

The essence of this mechanism is that the suppression of gene identity. The down-regulation of key negative regulatory transcription factors (e.g., WRKY25/33, JAZ) and signaling components (e.g., CaM/CML, CDPK) indicates that signal cascades were experienced synergistic inhibition. Overactivation of these cascades would lead to excessive defense responses, accelerated senescence, and growth arrest. This pattern implies that PGPR-HRW promotes a more energy-efficient stress response, allowing *P. giganteum* to maintain growth while withstanding salinity.

### 4.3 Molecular mechanisms of salt tolerance induced by PGPR-HRW combined treatment in *P. giganteum*

Through integrated transcriptomic and physiological findings, this study proposes that the synergistic action of PGPR-HRW induces a complex regulatory network centered on inhibiting key negative regulators. This network regulates salt tolerance through five interconnected mechanisms:

#### 4.3.1 Regulation of stomatal aperture by CaM/CML and ABA signaling

Salt stress typically triggers stomatal closure via ABA signaling to conserve water, at the cost of photosynthetic efficiency (Zhao et al., 2022; Hedrich and Shabala, 2018). Our data reveal a dual mechanism to maintain stomatal opening. First, exogenous H_2_ supplied via HRW significantly downregulated CaM/CML expression in *P. giganteum* (**Figure 7b**). This change was associated with reduced NOS activity and suppressed NO production in leaves, thereby alleviating NO-mediated stomatal closure. This aligns with previous reports of H_2_ modulating gas signaling and attenuating NO-dependent stress responses (Zhu et al., 2016) and HRW alleviated the inhibition of Al-induced root elongation in alfalfa by reducing NO production (Chen et al., 2014). Consistent with this mechanism, our study observed inhibition of stomatal closure under PGPR-HRW. Second, within the ABA signaling pathway, the upregulation of the negative regulator PP2C and the downregulation of the positive regulator SnRK2 synergistically suppressed the closure signal initiated by ABA (**Figure 5b**). This transcriptional pattern provides a direct pathway for stomata to maintain a better open state under PGPR-HRW treatment, considering ABA’s central role in the closure of stomata induced by drought/salt stress (Liu et al., 2022). Notably, 5 DEGs of negative regulatory factor PP2C were significantly up-regulated (A2B0 vs A0B0) explained the inherent salt tolerance of *P. giganteum* (**Figure 5b**), which is then significantly enhanced following PGPR-HRW treatment.

#### 4.3.2 Maintaining ROS balance and promoting defense response

When plants are subjected to salt stress, they need to activate ROS system to promote defense response, but excessive ROS would damage cells (Leshem et al., 2007). In physiological measurements of *P. giganteum*, PGPR-HRW under high salt stress (500 mM) significantly increased the activities of several antioxidant enzymes and the contents of osmotic regulatory substances, while decreasing levels of peroxidation products such as MDA and H_2_O_2_. (**Figure 2**). These physiological changes were consistent with reports that PGPR and HRW enhance plant antioxidant capacity (Giannelli et al., 2024). In KEGG enrichment analysis, two main pathways were found to be related to increased ROS system activity. In MAPK signaling pathway - plant, the negative regulatory factor CaM4 calmodulin was significantly down-regulated in all three comparison groups (**Figure 7a**), thereby maintaining ROS balance. Second, in the FLS2 pathway of Plant-pathogen interactions, the down-regulated expression of *WRKY25/33* DEGs, which function as negative transcriptional regulators (**Figure 7c**), enhanced the defense response of *P. giganteum* under salt stress. Previously, it was observed that the number of down-regulated genes in *P. giganteum* was greater than up-regulated genes in all three comparison groups (**Figures 3d–f**). *P. giganteum* likely alleviated salt stress effects primarily by inhibiting the expression of certain negative regulatory factors when subjected to salt stress or PGPR-HRW treatment. Overall, these changes release the suppression of antioxidant and defense genes, helping plants maintain reactive oxygen species at manageable levels while preserving their defense capabilities.

#### 4.3.3 Attenuating the HR to minimize cellular damage

The HR is a localized, programmed cell death (PCD) that can help defense, that assists defense mechanisms but causes tissue damage when overactivated under non-biotic stress (Lam et al., 2001; Baebler et al., 2020; Noman et al., 2020). Morphologically, leaves display browning, wilting, and other deteriorative states (Liu et al., 2022). Transcriptome data indicated that HR showed a significant downward trend under PGPR-HRW. First, calcium sensors and relays were suppressed. CaM/CML and CDPKs showed coordinated down-regulation (**Figures 6**, **7b**), which would weaken Ca^2+^-dependent signals that feed into HR execution. Second, expression of the RPM1/2 disease-resistant protein was also down-regulated (**Figure 6**), indicating that the NLR-mediated HR pathway was in a lower state of activation. These changes combined to reduce the occurrence of HR-induced programmed cell death under salt stress. The phenotypic changes in *P. giganteum* also confirmed this mechanism, that is, PGPR-HRW treatment reduced leaf yellowing, increased biomass, and mitigated HR response under high salt stress. Our results were highly consistent with Akbar’s findings. They inoculated two PGPR, *Bacillus subtilis* and *Bacillus pumilus*, into salt-stressed cotton and observed similar transcriptomic results, with down-regulation of genes related to CaM/CML, CDPK, and Rboh following PGPR salt stress treatment (Akbar et al., 2022).

#### 4.3.4 Inhibition of ethylene synthesis and delayed senescence

Regarding the response mechanism of ETH under salt stress, scientists have two differing views. Some suggest that ETH production enhances plant salt tolerance (Jahan et al., 2021; An et al., 2018), while others argue that ETH production accelerates plant senescence under stress (Paes de Melo et al., 2022; Gou et al., 2022). In our study, it was found that PGPR-HRW treatment inhibited ETH synthesis, delaying plant senescence under salt stress. In our previous study, it has been demonstrated that the employed PGPR-*C. firmus* L71 functions as an ACC deaminase-producing enzyme, which inhibits ETH production by catabolizing ETH precursors. At the transcriptome level, MPK3/6, as inducers of ethylene synthesis, cell death, and H_2_O_2_ production in MAPK signaling pathway-plant, showed down-regulated expression trend in all three comparison groups (**Figure 7d**). Additionally, in JA signaling of Plant hormone signal transduction pathway, significant down-regulation of JAZ DEGs inhibited plant senescence and stress response (**Figure 5c**), which together slowed down plant senescence. Our qRT-PCR results validated this. Among 12 selected DEGs, the first 8 were ETH activation signaling pathway/ETH response transcription factors (**Supplementary Table S3**), all observed low expression under A2B1 treatment (**Figure 8**). It was inferred that that delayed senescence is a key component of the PGPR-HRW induced tolerance.

#### 4.3.5 Promotion cytokinin synthesis

Cytokinin (CTK) acts as antagonistic hormone to ABA and ETH under salt stress, promoting cell division and inhibiting the senescence effects caused by ABA and ETH (Li et al., 2021). In CTK signaling pathway of Plant hormone signal transduction, high expression of regulatory factor A-ARR related to cell division and shoot initiation was observed in all three comparison groups (**Figure 5a**). This indicated that PGPR-HRW effectively promoted cell division and shoot initiation in *P. giganteum*, enhancing its response to salt stress. This may be related to the fact that RGPR inhibits ETH production, which is negatively feedback-regulated with CTK (Yu et al., 2023). The results highlight the interconnectedness of hormonal pathways in this coordinated response.

In summary, it was concluded that the molecular mechanisms by which PGPR-HRW combined treatment induced salt tolerance in *P. giganteum* primarily included the following five mechanisms: maintaining stomatal opening, maintaining ROS balance and promoting defense responses, inhibiting HR response, inhibiting ETH synthesis and delaying senescence, and promoting CTK synthesis. These mechanisms were not independent. Some DEGs functioned in different pathways, collectively forming the PGPR-HRW salt stress response network and required further study. Notably, certain key DEGs appearing in multiple pathways could alter plant salt tolerance when over-expressed or knocked out, providing new insights for breeding salt-tolerant plants.

This study revealed a novel integrated perspective on the synergistic interaction between PGPR and HRW at the molecular and physiological levels. The core mechanism revealed in this study is that this synergistic effect is due to the extensive and strategic suppression of negative regulators across multiple signaling pathways (MAPK pathway, hormone pathway, pathogen response pathway). This precise inhibitory regulation enables *P. giganteum* to more efficiently modulate its stress responses, thereby conserving energy and resources for growth and maintenance processes. From a practical application perspective, the PGPR-HRW combination demonstrated a highly promising sustainable strategy for phytoremediation and agricultural production in the saline soil. By combining the naturally salt-tolerant *P. giganteum* with a precisely formulated biological agents, the efficiency of the saline soil remediation could be significantly enhanced. The future research should focus on field trials to validate this synergistic effect under natural conditions and explore its application efficacy in other economically important and salt-tolerant crops.

## 5 Conclusion

This study demonstrated that the combined application of PGPR and HRW significantly enhanced the salt tolerance of *P. giganteum* under different NaCl levels. The treatment improved plant growth traits, including plant height, root length, and shoot biomass, while enhancing antioxidant enzyme activities and osmotic adjustment, and reducing oxidative damage. These effects became more pronounced under higher salinity, highlighting the potential of PGPR-HRW for severe salt stress mitigation. Transcriptomic analysis revealed that PGPR-HRW treatment primarily alleviated salt stress by suppressing negative regulators. Key down-regulated genes included CaM/CML, CaM4, CDPK, WRKY25/33, and JAZ. Enrichment of the “Plant hormone signal transduction”, “MAPK signaling”, and “Plant-pathogen interaction” pathways further supported the coordinated regulation of stress responses. Overall, the mechanisms underlying PGPR-HRW induced tolerance involve maintaining stomatal opening, restoring ROS balance, suppressing HR, reducing ethylene synthesis and delaying senescence, and promoting cytokinin signaling. These findings provide new insights into the interactive roles of microbial inoculants and HRW in plant stress tolerance. The identified pathways and candidate genes merit further functional validation, which may contribute to breeding and management strategies for enhancing crop resilience in saline environments.

## Data Availability

The datasets presented in this study can be found in online repositories. The names of the repository/repositories and accession number(s) can be found below: https://www.ncbi.nlm.nih.gov/, PRJNA1321525.
