# Supplementary material for: Synergistic alleviation effects of salt-tolerant plant growth-promoting rhizobacteria and hydrogen-rich water on salt stress in Pennisetum giganteum
Source: Front Plant Sci. 2025 Oct 29;16:1702577. doi: 10.3389/fpls.2025.1702577 (PMC12605088; doi:10.3389/fpls.2025.1702577)
Supplement: Supplementary file 1 [file DataSheet1.docx]

**Supporting Information**

**Synergistic alleviation effects of salt-tolerant plant growth-promoting rhizobacteria (PGPR) and hydrogen-rich water (HRW) on salt stress in *Pennisetum giganteum***

**Contents**

**Text S1.** RNA extraction

**Text S2.** Library preparation and Sequencing

**Text S3.** Quality control and De novo Assembly

**Text 4.** Differential expression analysis and Functional enrichment

**Fig. S1** Growth characteristics of *P. giganteum* between different NaCl and PGPR-HRW treatments

**Fig. S2** Physiological characteristics of *P. giganteum* between different NaCl and PGPR-HRW treatments

**Fig. S3** Salt and nutrient characteristics of *P. giganteum* between different NaCl and PGPR-HRW treatments

**Fig. S4** Combined score of *P. giganteum* between different NaCl and PGPR-HRW treatments based on principal component analysis (PCA)

**Table S1** Summary of read statistics from RNA-sequencing of *P. giganteum*

**Table S2** Comparison of sequencing data with assembly results from RNA-sequencing of *P. giganteum*

**Table S3** DEGs ID, annotation description (NR) and primer pairs for qRT-PCR analysis of 12 DEGs

**Text S1.** RNA extraction

Total RNA from *P. giganteum* leaves was extracted using TRIzol® Reagent. 3 replicates were set up for each treatment. RNA quality was determined with a 5300 Bioanalyser (Agilent) and quantified using the ND-2000 (NanoDrop Technologies). Only high-quality RNA samples (OD260/280 = 1.8-2.2, OD260/230 ≥ 2.0, RQN ≥ 6.5, 28S:18S ≥ 1.0, >1 μg) were used to construct the sequencing library.

**Text S2.** Library preparation and Sequencing

The *P. giganteum* RNA-seq transcriptome library was prepared using the Illumina® Stranded mRNA Prep, Ligation kit (San Diego, CA) with 1 μg of total RNA. In brief, messenger RNA was isolated using the polyA selection method with oligo(dT) beads and then fragmented using fragmentation buffer. Double-stranded cDNA was synthesized using a SuperScript double-stranded cDNA synthesis kit (Invitrogen, CA) with random hexamer primers. The synthesized cDNA was then subjected to end-repair, phosphorylation, and adapter addition following the library construction protocol. Libraries were size-selected for cDNA target fragments of 300 bp on 2% Low Range Ultra Agarose and then PCR amplified using Phusion DNA polymerase (NEB) for 15 cycles. After quantification by Qubit 4.0, the sequencing library was sequenced on the NovaSeq X Plus platform (PE150) using the NovaSeq Reagent Kit or on the DNBSEQ-T7 platform (PE150) using the DNBSEQ-T7RS Reagent Kit (FCL PE150) version 3.0.

**Text S3.** Quality control and De novo Assembly

The raw paired-end reads were trimmed and quality-controlled using fastp with default parameters. Clean data from the samples were then used for de novo assembly with Trinity. To enhance assembly quality, all assembled sequences were filtered with CD-HIT and TransRate and assessed using BUSCO (Benchmarking Universal Single-Copy Orthologs).

**Text 4.** Differential expression analysis and Functional enrichment

To identify differentially expressed genes (DEGs) between two different samples/groups, the expression level of each transcript was calculated using the transcripts per million reads (TPM) method. RSEM was employed to quantify gene abundances. Differential expression analysis was performed using DESeq2. DEGs with |log2FC| ≥ 1 and FDR < 0.05 (DESeq2) were considered significantly differentially expressed. Additionally, functional enrichment analysis using the Kyoto Encyclopedia of Genes and Genomes (KEGG) was conducted to determine which DEGs were significantly enriched in KEGG pathway analysis, performed with Python's SciPy library. Diamond was used to identify proteins with the highest sequence similarity to the given transcripts for functional annotations, with a typical cut-off E-value of less than 1.0 × 10^−5^.

**
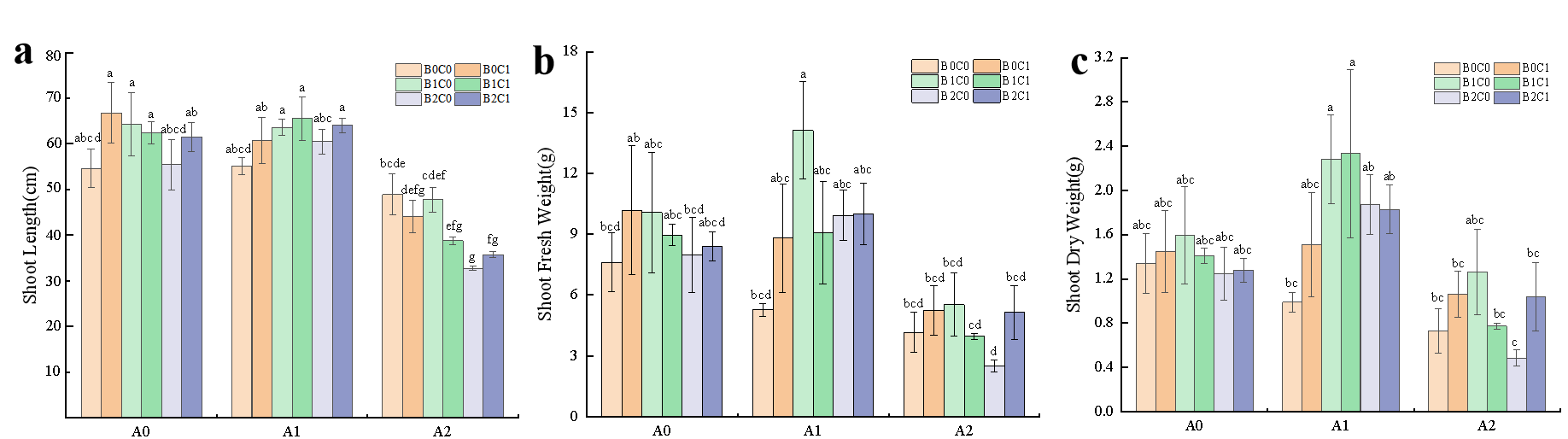
Fig. S1** Growth characteristics of *P. giganteum* between different NaCl and PGPR-HRW treatments

**Note:** A0: 0mM NaCl, A1:250mM NaCl, A2:500mM NaCl; B0: 0% HRW, B1: 50% HRW, B2: 100% HRW; C0:no *C.firmus* L71 added, C1: *C.firmus* L71 added. (a) Shoot length, (b) Shoot fresh weight (c) Shoot dry weight. Different lowercase letters indicate significant differences between treatments (*p*＜0.05).

**
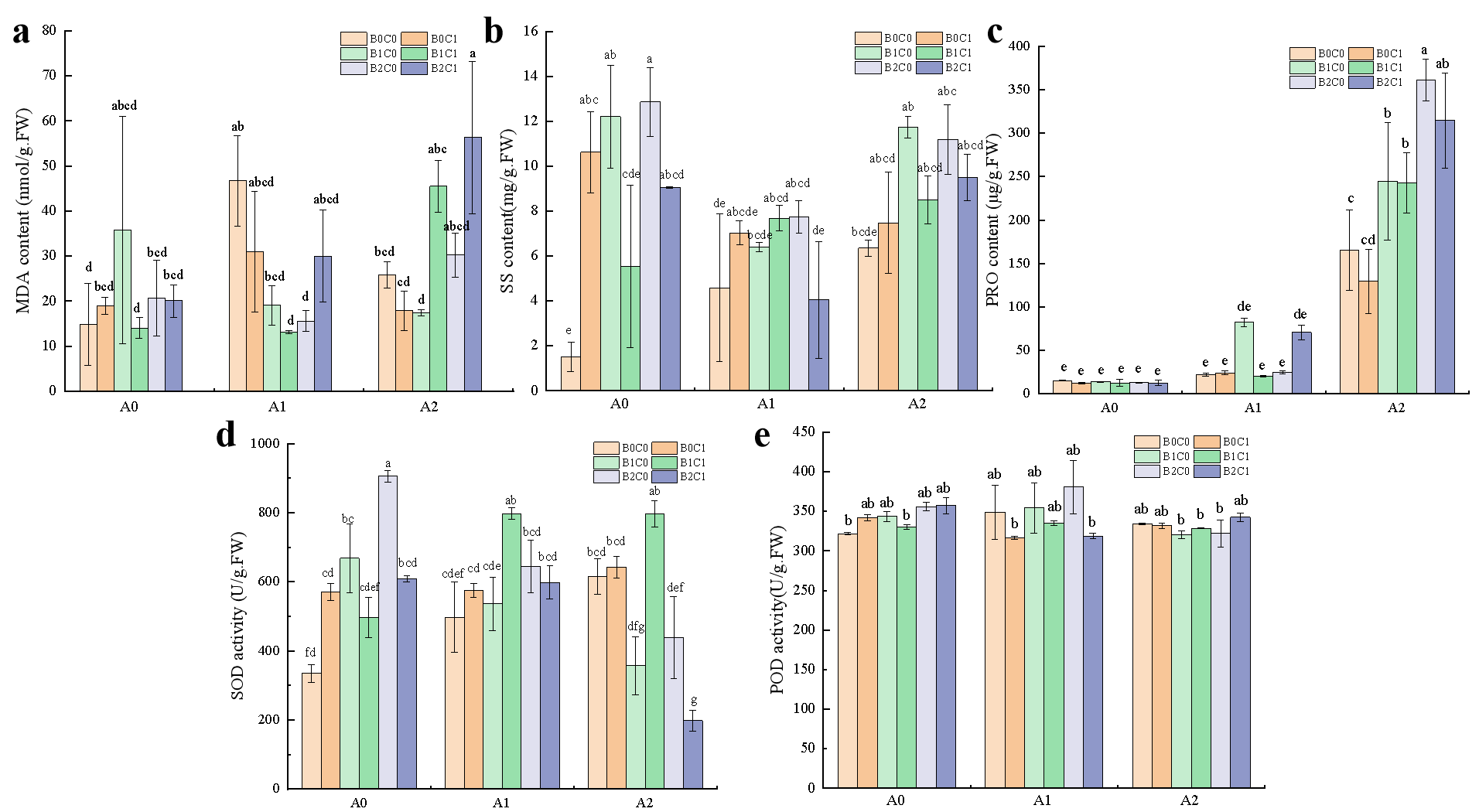
Fig. S2** Physiological characteristics of *P. giganteum* between different NaCl and PGPR-HRW treatments

**Note:** A0: 0mM NaCl, A1:250mM NaCl, A2:500mM NaCl; B0: 0% HRW, B1: 50% HRW, B2: 100% HRW; C0:no *C.firmus* L71 added, C1: *C.firmus* L71 added. (a): MDA, (b): SS, (c) Pro contents. (d): SOD, (e): POD activities. Different lowercase letters indicate significant differences between treatments (*p*＜0.05).

**Fig. S3** Salt and nutrient characteristics of *P. giganteum* between different NaCl and PGPR-HRW treatments

**Note:** A0: 0mM NaCl, A1:250mM NaCl, A2:500mM NaCl; B0: 0% HRW, B1: 50% HRW, B2: 100% HRW; C0:no *C.firmus* L71 added, C1: *C.firmus* L71 added. (a-c) Shoot K^+^(a), Na^+^(b) K^+^/Na^+^(c), (d-f) Root K^+^(d), Na^+^(e) and K^+^/Na^+^(f). Different lowercase letters indicate significant differences between treatments (*p*<0.05), and different capital letters indicate significant differences between treatments of the same salt stress concentration (*p*<0.05).

**Fig. S4** Combined score of *P. giganteum* between different NaCl and PGPR-HRW treatments based on principal component analysis (PCA)


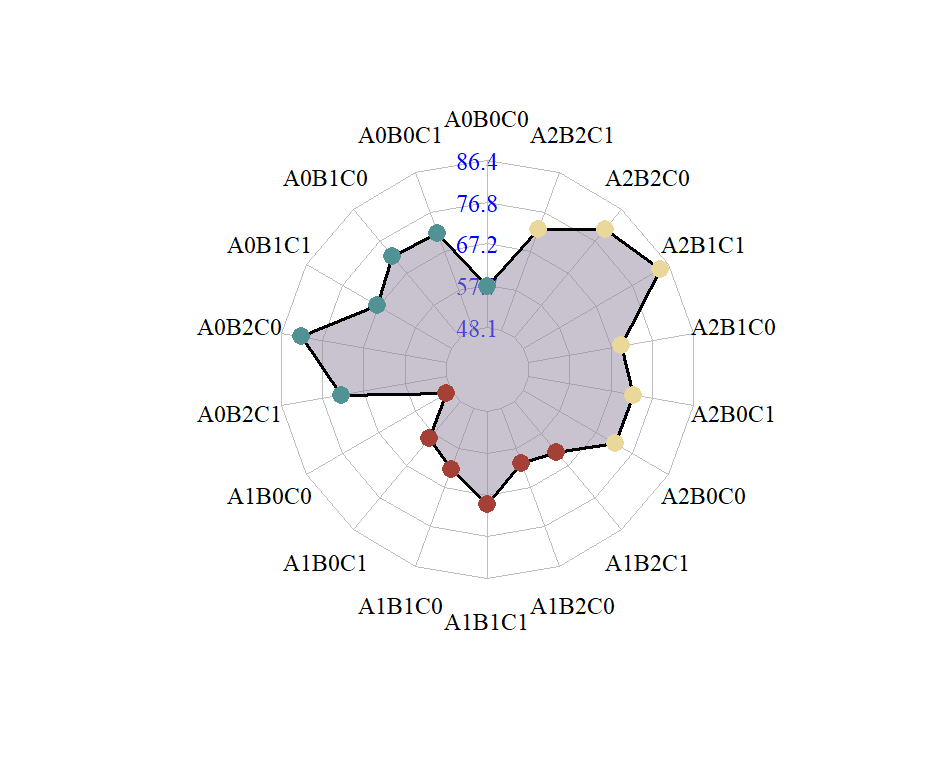


**Note:** Total of 12 indicators, including plant height, shoot dry weight and fresh weight, MDA content, SS content, Pro content, SOD and POD activity, shoot K^+^ content and K^+^/Na^+^ratio, root K^+^content and K^+^/Na^+^ratio, were used to carry out principal component analysis (PCA) and weighting analysis for the 18 treatment combinations under different salt stresses. Before the PCA, the mean values of the indexes of each treatment combination were z-standardized.

**Table S1** Summary of read statistics from RNA-sequencing of *P. giganteum*

| Sample | Raw reads | Raw bases | Clean reads | Clean bases | Error rate (%) | Q20(%) | Q30(%) | GC content (%) |
| --- | --- | --- | --- | --- | --- | --- | --- | --- |
| A0B0_1 | 52148402 | 7874408702 | 51808582 | 7756918863 | 0.012 | 98.72 | 95.92 | 49.9 |
| A0B0_2 | 40144286 | 6061787186 | 39867992 | 5979275418 | 0.012 | 98.74 | 95.98 | 49.62 |
| A0B0_3 | 47109610 | 7113551110 | 46781532 | 7016818213 | 0.012 | 98.75 | 96.02 | 49.72 |
| A0B1_1 | 46242212 | 6982574012 | 45937228 | 6892838030 | 0.012 | 98.75 | 96.02 | 49.6 |
| A0B1_2 | 50971924 | 7696760524 | 50647100 | 7588165186 | 0.0119 | 98.8 | 96.18 | 49.36 |
| A0B1_3 | 48672218 | 7349504918 | 48353280 | 7255969035 | 0.012 | 98.76 | 96.05 | 49.17 |
| A2B0_1 | 43837228 | 6619421428 | 43533196 | 6525603392 | 0.012 | 98.77 | 96.08 | 49.36 |
| A2B0_2 | 48787238 | 7366872938 | 48460902 | 7251378273 | 0.0119 | 98.78 | 96.12 | 49.15 |
| A2B0_3 | 50892472 | 7684763272 | 50563390 | 7562671361 | 0.0119 | 98.8 | 96.18 | 48.95 |
| A2B1_1 | 58333468 | 8808353668 | 57974562 | 8690320841 | 0.0119 | 98.8 | 96.19 | 49.22 |
| A2B1_2 | 56256854 | 8494784954 | 55900152 | 8386165374 | 0.0119 | 98.78 | 96.13 | 49.14 |
| A2B1_3 | 51881616 | 7834124016 | 51569986 | 7737307802 | 0.0119 | 98.81 | 96.2 | 48.55 |

**Notes:** Raw reads: The total number of original sequencing entries. Raw bases: The total amount of original sequencing data. Clean reads: The total number of sequencing entries after quality control. Clean bases: The total amount of sequencing data after quality control. Error rate (%): The average base error rate corresponding to the quality-controlled data. Q20 (%), Q30 (%): Quality assessment of the sequencing data after quality control, with Q20 and Q30 representing the percentage of bases with sequencing quality above 99% and 99.9%. GC content (%): The percentage of G and C bases in the total bases corresponding to the quality-controlled data.

**Table S2** Comparison of sequencing data with assembly results from RNA-sequencing of *P. giganteum*

| Sample | Clean reads | Mapped reads | Mapped ratio |
| --- | --- | --- | --- |
| A0B0_1 | 25904291 | 22174477 | 85.60% |
| A0B0_2 | 19933996 | 17100111 | 85.78% |
| A0B0_3 | 23390766 | 19885518 | 85.01% |
| A0B1_1 | 22968614 | 19687699 | 85.72% |
| A0B1_2 | 25323550 | 21550247 | 85.10% |
| A0B1_3 | 24176640 | 20558109 | 85.03% |
| A2B0_1 | 21766598 | 18687620 | 85.85% |
| A2B0_2 | 24230451 | 20787809 | 85.79% |
| A2B0_3 | 25281695 | 21674645 | 85.73% |
| A2B1_1 | 28987281 | 24938266 | 86.03% |
| A2B1_2 | 27950076 | 23983270 | 85.81% |
| A2B1_3 | 25784993 | 21920240 | 85.01% |

**Notes**: Clean reads (pair reads): The number of sequencing entries after filtering. Mapped reads: The number of clean reads (pair reads) that can be aligned to the assembled transcripts. Mapped ratio: The percentage of clean reads (pair reads) that can be mapped to the assembled transcripts.

**Table S3** DEGs ID, annotation description (NR) and primer pairs for qRT-PCR analysis of 12 DEGs

| DEGs ID | Annotation description (NR) | Forward primer（5'-3'） | Reverse primer（5'-3'） |
| --- | --- | --- | --- |
| Actin (GAPDH) |  | CCATCACTGCCACACAGAAAAC | AGGAACACGGAAGGACATACCAG |
| TRINITY_DN3113_c0_g1 | ethylene-responsive transcription factor 4-like [Panicum hallii] | ACCAACGCAACAACAACAACAACC | ACGACGGCGGGACGAAGC |
| TRINITY_DN10053_c0_g1 | hypothetical protein EJB05_09752, partial [Eragrostis curvula] | AAGAAGCCGAGAACGCAAACCC | TGCCTGCCTGTAAGGACTATGGAG |
| TRINITY_DN18187_c0_g3 | ethylene-responsive transcription factor 11 [Setaria italica] | ACGGCAGGCAAGCAAGCAAG | GCGCCATGGTATCCCAAGAACC |
| TRINITY_DN13156_c1_g1 | ethylene-responsive transcription factor 4-like [Setaria viridis] | GCCTGCTCGATCCTGTTGTATTCC | TTGCCGCTGGGTTTGTGTTCTC |
| TRINITY_DN4345_c1_g2 | ethylene-responsive transcription factor ERF105 [Setaria italica] | CTGCCACCGCTGGTAGTAACAAG | GCTTGCGACTGGCTCCTGAAC |
| TRINITY_DN4461_c0_g1 | hypothetical protein GQ55_6G068100 [Panicum hallii var. hallii] | CGTCGTCCTGCCAGATGTTCTTG | TGCTCCTTCTCCTCGCCCTTC |
| TRINITY_DN4461_c0_g2 | hypothetical protein PVAP13_4NG229511 [Panicum virgatum] | GGTTTGGCAACCTTGTGCATTGAG | CTCCTGGATGCAAAGTCGGTGATG |
| TRINITY_DN4345_c1_g3 | ethylene-responsive transcription factor ERF105-like [Setaria viridis] | GCAGCAGCAGCAGCAGCAG | CGGAAGTCGTCCTCCTCAGGTC |
| TRINITY_DN3969_c0_g1 | ethylene-responsive transcription factor 11 [Setaria italica] | ACCACGTCGCTGGACCTGAG | TGCTGATGATCCTCCTCGCTCTTC |
| TRINITY_DN10967_c0_g1 | NAC domain-containing protein 21/22 [Dichanthelium oligosanthes] | CGGCGGCAAGGTCGGATTC | CGGTCCTTGAGGCTGAAGAAGTAC |
| TRINITY_DN50304_c0_g1 | protein DETOXIFICATION 45, chloroplastic [Setaria italica] | ACAGTGGTTTGGGCAACATCTCAG | AAGTGCGGCTCCAGTTAATCCAAG |
| TRINITY_DN76994_c0_g1 | probable glutathione S-transferase GSTU6 [Panicum virgatum] | GTCCTGATCCACAACGGCAACC | CGGCAGCGGAAGGGAAAGC |
